# Lower limb ice application alters ground reaction force during gait initiation

**DOI:** 10.1590/bjpt-rbf.2014.0080

**Published:** 2015-04-27

**Authors:** Thiago B. Muniz, Renato Moraes, Rinaldo R. J. Guirro

**Affiliations:** 1 Centro de Reabilitação, Hospital das Clínicas, Faculdade de Medicina de Ribeirão Preto (FMRP), Universidade de São Paulo (USP), Ribeirão Preto, SP, Brazil; 2 Escola de Educação Física e Esporte de Ribeirão Preto, USP, Ribeirão Preto, SP, Brazil; 3 Departamento de Biomecânica, Medicina e Reabilitação do Aparelho Locomotor, FMRP, USP, Ribeirão Preto, SP, Brazil; 4 Programa de Pós-graduação em Reabilitação e Desempenho Funcional, FMRP, USP, Ribeirão Preto, SP, Brazil

**Keywords:** physical therapy, gait, neuromuscular control, force platform, cryotherapy

## Abstract

**BACKGROUND:**

Cryotherapy is a widely used technique in physical therapy clinics and sports. However, the effects of cryotherapy on dynamic neuromuscular control are incompletely explained.

**OBJECTIVES:**

To evaluate the effects of cryotherapy applied to the calf, ankle and sole of the foot in healthy young adults on ground reaction forces during gait initiation.

**METHOD:**

This study evaluated the gait initiation forces, maximum propulsion, braking forces and impulses of 21 women volunteers through a force platform, which provided maximum and minimum ground reaction force values. To assess the effects of cooling, the task - gait initiation - was performed before ice application, immediately after and 30 minutes after removal of the ice pack. Ice was randomly applied on separate days to the calf, ankle and sole of the foot of the participants.

**RESULTS:**

It was demonstrated that ice application for 30 minutes to the sole of the foot and calf resulted in significant changes in the vertical force variables, which returned to their pre-application values 30 minutes after the removal of the ice pack. Ice application to the ankle only reduced propulsion impulse.

**CONCLUSIONS:**

These results suggest that although caution is necessary when performing activities that require good gait control, the application of ice to the ankle, sole of the foot or calf in 30-minute intervals may be safe even preceding such activities.

## Introduction

Although cryotherapy is widely used in clinical practice, its associated physiological responses have not been fully explained^1^. In addition to reducing pain, cryotherapy has been reported to promote other changes^2^, such as reduced nerve conduction velocity^3^^,^ 4, inhibition of nociceptors and muscle spasm reduction^5^.

Uchio et al.^6^ noted a concern with resuming exercise after cryotherapy. For example, Ribeiro et al.^7^ noted that the application of ice for 30 minutes to the knee joint reduced joint position sense. Hopper et al.^8^ also observed a reduction in ankle joint position sense after 15 minutes of ice application to this joint.

The maintenance of balance involves several integrated body systems, including the musculoskeletal and proprioceptive systems as well as other sensory systems (e.g., vision and vestibular) and central integration processes^9^. Previous studies on joint position sense have indicated that proprioception, which includes information from muscle, skin and joint receptors^10^, is particularly affected by cryotherapy^8^. Thus, cryotherapy may compromise the sensorimotor system by changing the response of these receptors^11^. Because the sensorimotor system is essential for joint stability and protection, any intervention that impairs its proper functioning may predispose an individual to injury.

Therefore, the present study evaluated the ground reaction force (GRF) during a dynamic task, gait initiation, following ice application to different structures - sole of the foot, ankle and triceps surae (commonly known as the calf muscles). These three regions were chosen for their predominance of skin, joint and muscle receptors, respectively, which might differentially influence gait initiation following cryotherapy. In addition, it is known that the ankle and calf are commonly injured during sports practice^12^^,^ 13 and that the sole of the foot possesses mechanoreceptors that are essential to the stability of the stance phase of the gait cycle^14^. Therefore, an assessment of the effects of ice on these three regions is of considerable clinical relevance.

## Method

### Sample

In total, 21 young adult female university students were randomly recruited from the *Faculdade de Medicina de Ribeirão Preto, Universidade de São Paulo* (FMRP-USP), Ribeirão Preto, state of São Paulo - SP, Brazil. Women were recruited because the lack of gender differences in the effects of ice application^15^ has been established in the literature and due to ease of recruiting. To be included in the study, the volunteer participants were required to be healthy and sedentary, have no history of lower limb sensory nerve damage, musculoskeletal injury, trauma or skin lesions, and not be in the week preceding a menstrual period or to be menstruating due to the sensory changes associated with this period^16^. This project was approved by the Research Ethics Committee of the FMRP-USP (protocol no. 635/2009), and all participants signed an informed consent form.

### Instruments

An OR 6-7-1000 force plate (AMTI, Watertown, MA, USA) with a sampling frequency of 100 Hz was used to record the forces applied to the ground. An ST-600 infrared digital thermometer with laser sight (Incoterm(r), Porto Alegre, State of Rio Grande do Sul - RS, Brazil) with a temperature reading range from -60 to 500 °C, precision of ± 2% and resolution of 0.1 °C was used to measure the skin temperature of the evaluated regions. To standardize the pressure exerted during ice application, a sphygmomanometer was used (*BD*(r), Juiz de Fora, State of Minas Gerais - MG, Brazil) with the cuff positioned between the ice pack and strap at a constant pressure of 30 mmHg^17^. An EGE-300M ice machine (Everest(r), Rio de Janeiro, State of Rio de Janeiro - RJ, Brazil) with a production capacity of 300 kg of ice per day and 100 kg storage capacity at a temperature of 0.5°C was utilized to produce and store the ice.

### Procedures

Following a standard physical examination, the order of ice application sites for the volunteers was randomly drawn by lot. Each subject underwent ice application to the three regions on three different days with a minimum interval of 48 hours between applications, and each ice application session lasted 30 minutes^18^.

Each day, ice was applied to the non-dominant limb, to one of the three target areas (i.e., sole, ankle or calf), at pre-scheduled morning appointments, and data was collected in a climate-controlled room (23 °C±2 °C). The data were collected before ice application (Pre), immediately after the ice was removed (T0) and 30 minutes after the ice was removed (T30).

The ice was applied using a plastic bag containing 1.5 kg of crushed ice in direct contact with the skin of the subject. To apply the ice, the subjects were seated^15^ with their knees and hips positioned at 90°. Before each assessment (Pre, T0 or T30), skin temperature was recorded using the infrared digital thermometer with laser sight, which was positioned perpendicular to the segment, 15 cm away from the skin. Multiple measurements were taken from different segment positions to improve estimates of the actual temperature. The following locations were measured: three plantar points (i.e. center of the forefoot, midfoot and hindfoot); four ankle points (i.e. center of each malleoli and anterior and posterior middle points of the segment between them); and three calf points (i.e. center of the proximal, middle and distal third). The mean temperatures of the different points measured for each segment were used.

The task evaluated in this study was gait initiation. For this purpose, the volunteers remained in the initial position (i.e. an upright, quiet position with feet parallel and spaced shoulder width apart) and were instructed to perform the first step with the non-dominant limb on the force plate. Dominance was assessed through the task of kicking a ball. The data collected for this part of the study refers to the initial step performed unilaterally with the non-dominant limb. To perform the step, the force plate was fixed between two wooden platforms of the same height and width at the same level. The volunteers were instructed to perform the step and walk as naturally as possible; therefore, the speed was not standardized. After the tests, subjects remained seated with the heel resting on a wooden surface to prevent heat exchange.

### Data analysis

**Figure 1.**
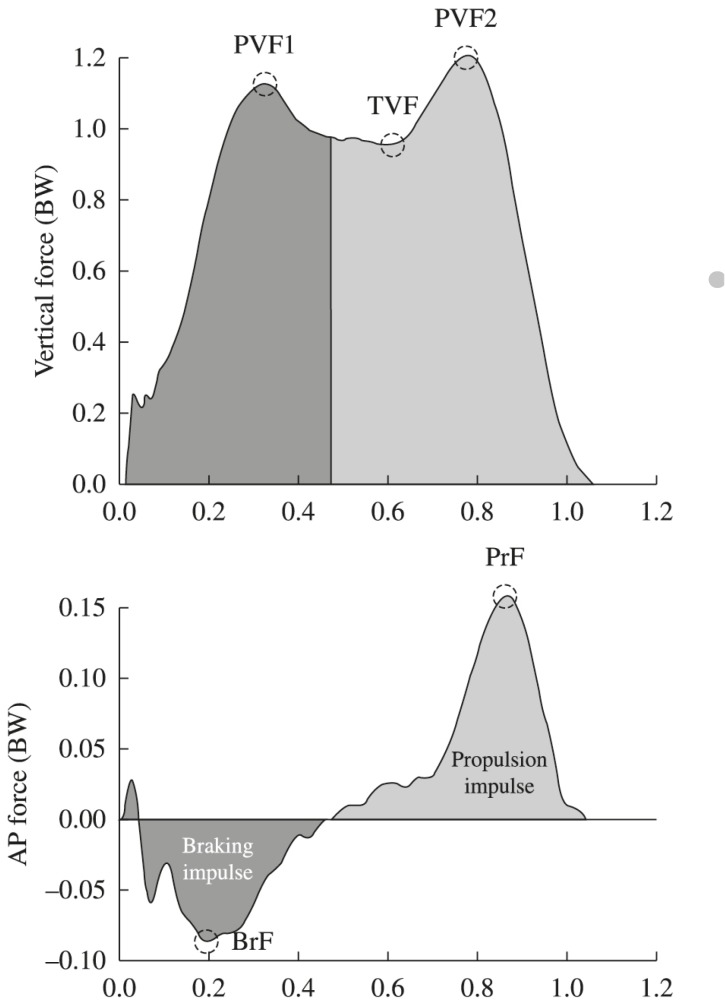
Force-time curves for the vertical (top) and anteriorposterior (AP, bottom) directions. Dark shaded areas correspond to the braking impulse; light shaded areas illustrate the propulsive impulse. PVF1: first peak of the force in the vertical direction; PVF2: second peak of the force in the vertical direction; TVF: valley of the force in the vertical direction; BrF: braking force in the AP direction; PrF: propulsion force in the AP direction; BW: body weight.

The anteroposterior (Fap) and vertical components (Fv) of GRF were analyzed in this study. The GRF values were normalized by subject body weight (BW). After normalization, the following variables were calculated in the vertical direction (Figure 1): peak value immediately after contact of the foot with the ground (PVF1), peak value before lifting the foot from the ground (PVF2) and valley value between peaks 1 and 2 (TVF). In the anteroposterior direction (AP), the maximum braking force (BrF) and maximum propulsive force (PrF) were calculated. In addition to these variables, the braking and propulsive impulses in the vertical and AP directions were calculated^19^. The force curve in the AP direction was used to identify the transition between braking and propulsion as the instant the curve crossed the zero axis (Figure 1). The braking impulse was calculated as the area under the force-time curve from foot contact with the platform (Fv≥5 N) until the moment that the curve crossed the zero axis, and the propulsive impulse was calculated from that moment until the foot was lifted from the force plate (Fv≤5 N).

Based on the GRF, the durations of the following gait phases were also calculated: stance phase (SD), braking phase (BD) and propulsion phase (PD). The SD was calculated as the interval between contact and removal of the foot from the force plate. The BD was calculated as the interval between contact of the foot with the force plate and the instant that the Fap crossed the zero axis. The PD was measured as the interval between the instant that the Fap crossed the zero axis and removal of the foot from the ground.

### Statistical analysis

After tabulating the results, a Shapiro-Wilk normality test was applied to all variables. For variables with normal distributions, an ANOVA test for repeated measures and Tukey's test were applied. For data that were not normally distributed, a Kruskal-Wallis test and Dunn's test were utilized. The data were processed using SPSS 20.0 software (IBM, Armonk, New York, USA) and significance was set at a level of 5% (p<0.05).

Power was calculated by considering the vertical force with ice applied to the calf, and 95% power for T0 and 77% for T30 were obtained.

## Results

### Characteristics of the sample

The participants had the following characteristics: age of 21.7±1.9 years, body mass of 57.9±7.1 kg, height of 1.65±0.07 m and body mass index (BMI) of 21.4±2.1 kg/m^2^.

### Temperature

A significant reduction in skin temperature was observed immediately following ice pack removal (T0) to the three target areas (Table 1). Temperatures remained below their pre-application values after 30 minutes, although they had increased since T0.

**Table 1.** Mean values and standard deviations (±) of skin temperature for ice application sites (foot sole, ankle and calf) before (Pre), immediately after (T0) and 30 minutes after application (T30). n=21.

Site of ice application	Measurement Time		
	Pre	T0	T30
Foot Sole (°C)	27.57±1.5	7.84±1.74*	21.94±1.84*^#^
Ankle (°C)	29.36±1.05	10.69±2.45*	21.53±1.29*^#^
Calf (°C)	29.34±2.14	9.03±3.2*	25.11±1.44*^#^

* p=0.05 compared to Pre.

# p=0.05 compared to T0. Pre: temperature prior to application of ice; T0: temperature as soon as ice was removed; T30: temperature 30 minutes after ice was removed.

### Duration of the gait phases

Ice application did not affect the durations of the gait phases (Table 2). Overall, the duration of the stance phase was approximately one second, and participants spent more time braking than accelerating their bodies.

**Table 2.** Mean values and standard deviations (±) of support (SD), braking (BD) and propulsion (PD) durations (in seconds [s]) for the ice application sites (sole, ankle and calf) before application of ice (Pre), immediately after application of ice (T0) and 30 minutes after removal of ice (T30). n=21.

Site of ice application	Measurement Period		
	Pre	T0	T30
SD – Foot Sole (s)	1.018±0.109	1.035±0.109	1.041±0.108
SD – Ankle (s)	1.028±0.086	1.033±0.083	1.029±0.094
SD – Calf (s)	1.190±0.109	1.041±0.093	1.214±0.108
BD – Foot Sole (s)	0.560±0.131	0.587±0.083	0.593±0.094
BD – Ankle (s)	0.572±0.086	0.578±0.093	0.575±0.073
BD – Calf (s)	0.589±0.085	0.581±0.083	0.574±0.106
PD – Foot Sole (s)	0.459±0.111	0.448±0.080	0.448±0.086
PD – Ankle (s)	0.457±0.088	0.447±0.099	0.455±0.086
PD – Calf (s)	0.435±0.070	0.460±0.088	0.472±0.099

### Maximum and minimum GRF values

The application of ice to the sole and calf increased PVF1 immediately following application (Table 3). The same trend was observed for TVF. In addition, the values of these two variables were similar for Pre and T30 but decreased significantly between T0 and T30 for TVF for all sites and for PVF1 in the calf. For PVF2, there was a decrease in the values of these variables from Pre and T0 to T30 when the ice was applied to the ankle and calf. In the AP direction, BrF increased at T30 when compared to Pre with the application of ice to the ankle and sole of the foot. Conversely, PrF decreased at T30 compared to Pre with the application of ice to the ankle and calf. In addition, PrF decreased at T30 compared to T0 when ice was applied to the ankle.

**Table 3.** Mean values and standard deviations (±) of the vertical and anterior-posterior (AP) components of Ground Reaction Force for the ice application sites before application of ice (Pre), immediately after application of ice (T0), and 30 minutes after removal of ice (T30).

	Measurement Period		
	Pre	T0	T30
Vertical Component of GRF			
PVF1 – Foot Sole (BW)	1.020±0.042	1.035±0.049*	1.022±0.039
PVF1 – Ankle (BW)	1.024±0.047	1.027±0.042	1.024±0.046
PVF1 – Calf (BW)	1.020±0.045	1.030±0.045*	1.017±0.039^#^
TVF – Foot Sole (BW)	0.890±0.024	0.907±0.031*	0.890±0.026^#^
TVF – Ankle (BW)	0.892±0.027	0.900±0.030	0.887±0.026^#^
TVF – Calf (BW)	0.888±0.025	0.901±0.024*	0.886±0.024^#^
PVF2 – Foot Sole (BW)	1.050±0.037	1.034±0.054	1.036±0.037
PVF2 – Ankle (BW)	1.050±0.037	1.045±0.034	1.035±0.035*^#^
PVF2 – Calf (BW)	1.046±0.035	1.042±0.044	1.026±0.040*^#^
Anteroposterior Component of GRF			
BrF – Foot Sole (BW)	–0.083±0.027	–0.083±0.023	–0.088±0.025*
BrF – Ankle (BW)	–0.084±0.018	–0.091±0.030	–0.091±0.022*
BrF – Calf (BW)	–0.085±0.021	–0.084±0.021	–0.089±0.024
PrF – Foot Sole (BW)	0.117±0.027	0.110±0.026	0.107±0.024
PrF – Ankle (BW)	0.114±0.030	0.114±0.026	0.104±0.027*^#^
PrF – Calf (BW)	0.117±0.025	0.111±0.026	0.108±0.025*

* Significantly different from instant Pre (p<0,05).

# Significantly different from instant T0 (p<0,05). PVF1: first peak of the force in the vertical direction; PVF2: second peak of the force in the vertical direction; TVF: valley of the force in the vertical direction; BrF: braking force in the AP direction; PrF: propulsion force in the AP direction; BW: body weight.

### Impulses

**Figure 2.**
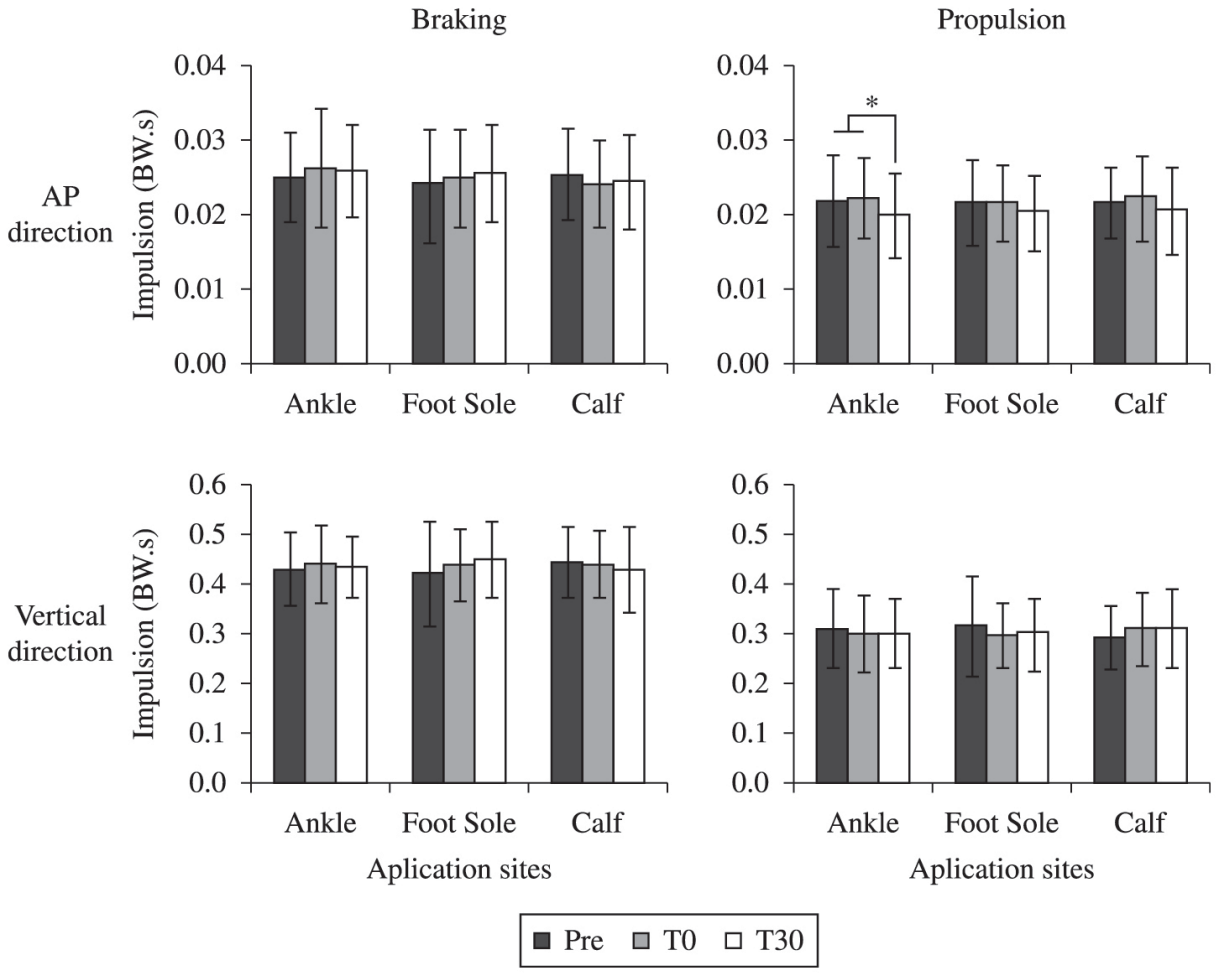
Means and standard deviations for braking (left side) and propulsive (right side) impulses in both anterior-posterior (AP, top) and vertical (bottom) directions for all ice application sites at three measurement times (Pre - prior to application of ice, T0 - immediately after the application of ice, and T30 - 30 minutes after the removal of ice). *p=0.05.

The application of ice to the ankle reduced the propulsive impulse in the AP direction at T30 compared to Pre and T0 (Figure 2).

## Discussion

Although skin temperature poorly predicts intramuscular temperature^20^, this parameter was chosen as a noninvasive method that is strongly correlated with nerve conduction velocity^21^, an extremely important variable for sensorimotor control. Thus, the results indicate that even 30 minutes after ice was removed, skin temperatures had not returned to baseline values. These data are consistent with previous research, which indicate that the skin temperatures of the target regions remained below pre-application values even after 60 minutes^22^^,^ 23.

An important result of this study was the observed increase in PVF1 immediately following ice application (T0) to the sole of the foot and calf. This result suggests increased overload on the musculoskeletal system immediately after foot contact with the ground. This overload can be partly explained by the fact that decreased temperatures affect neuromuscular system functioning, and both afferent and efferent nerve impulses might have slowed after cooling^24^.

Similarly, cooling the sole of the foot with ice has been previously described as responsible for increased weight transfer rates following foot contact with the ground at the end of the gait cycle^25^. Sensorimotor alterations caused by the application of ice might result from increased depolarization thresholds of plantar receptors^15^ as well as decreased firing rates of these receptors^26^. These changes tend to reduce afferent impulses to the central nervous system resulting in reduced limb control during the gait stance phase. Additionally, the decrease in calf temperature decreases the efferent impulses of this muscle and increases muscle rigidity^27^. According to Mustalampi et al.^28^, the application of ice to the quadriceps muscle temporarily increased rigidity and tension of the muscle as well as reducing its elasticity. Therefore, we suggest that the same pattern occurs in calf muscles, which generated lower impact absorption values when the hindfoot made contact with the force plate. Thus, both the reduction of afferent impulses to plantar and calf mechanoreceptors and increased rigidity of calf muscles might explain increased PVF1.

However, 30 minutes after the ice was removed from the sole of the foot and calf, PVF1 values returned to baseline levels despite that temperatures remained below pre-application levels. This result can be explained by the fact that decreased afferent impulses following cooling are quickly compensated by the central nervous system^28^. According to Billot et al.^29^, the central nervous system redistributes balance maintenance tasks to other sensory structures, which compensates for the deficit in afferent impulses from plantar mechanoreceptors.

The results indicate a reduction of the PVF2 peak after 30 minutes of ice application to the ankle and calf. This result suggests that even with reduced skin temperatures, the adaptations in the central nervous system allowed learning of the gait initiation task. The same pattern was not observed when ice was applied to the sole of the foot, which suggests the inability of the central nervous system to learn the required task due to the sensory losses caused by decreased afferent impulses from the plantar mechanoreceptors.

No changes in the variables studied were observed at time T0 following ice application to the ankle suggests that sensory information from this joint was not affected by reduced skin temperature. Although Hopper et al.^8^ observed a reduction in joint proprioception after reducing ankle temperatures, their results did not produce significant clinical values. The authors observed an angular change of only 0.5° immediately after ice removal. In addition, other studies have not observed changes in ankle joint proprioception after ice application^30^^,^ 31.

The reduction of ankle temperature might not change the GRF at T0 due to compensation from afferent impulses from mechanoreceptors of other regions. This hypothesis has been supported by observations that reduction of shoulder temperature does not change joint position sense^32^. These authors argue that sensory information from other peripheral areas might compensate for the afferent impulse deficit caused by applying ice to the joint. In addition, the application of ice might have been insufficient to reduce the temperature of deep joint mechanoreceptors and induce changes in GRF^33.^

At time T30, however, the application of ice to different body regions affected propulsion and braking values in the AP direction (BrF and PrF) and propulsive impulses. However, we can attribute these changes to learning because they were only observed during the last repetition (T30) when the temperature, although inferior to pre-application levels, does not seem to directly influence the functioning of the sensorimotor system.

Regarding the limitations of this study, the fact that direct measurements of the proprioceptive system were not performed, the absence of measurement of other variables and the assessment of other tasks do not allow conclusive statements about the effects of ice application on the proprioceptive system. However, the changes observed in this study and in the extant literature allow us to state that in the situations previously described, ice application was sufficient to cause sensorimotor changes during the gait stance phase. Although the task chosen - gait initiation - might limit the application of these results to other activities that require greater neuromuscular control (e.g. jumps, spins, acceleration and deceleration), in rehabilitation, one of the most important tasks is the reestablishment of natural gait. Thus, clarifying whether ice application, which is commonly used in clinical practice for ankle and calf injuries, can change gait is of considerable importance in rehabilitation settings.

## Conclusion

Cryotherapy predominately affected the load applied to the body after foot contact with the ground, particularly after ice application to the sole of the foot and calf in sedentary young adult women. These results suggest that although there is the need for caution when performing activities that require good gait control, ice application to the ankle or at a 30-minute interval after ice has been removed can be safe, even when preceding such activities.
